# Cytomegalovirus colitis as intestinal obstruction in an immunocompetent adolescent: a case report and literature review

**DOI:** 10.1186/s12879-024-09255-7

**Published:** 2024-04-01

**Authors:** Jiongshan Ge, Yan Li, Di Shi, Jiaxin Wei, Jing Wang, Jihai Liu

**Affiliations:** 1 Emergency Department, The State Key Laboratory for Complex, Severe and Rare Diseases, Peking Union Medical College Hospital, Chinese Academy of Medical Science and Peking Union Medical College, No. 1 Shuaifuyuan Wangfujing Dongcheng District, Beijing, 100730 China; 2 Pathology Department, Peking Union Medical College Hospital, Chinese Academy of Medical Science and Peking Union Medical College, No.1 Shuaifuyuan Wangfujing Dongcheng Districtg, Beijing, 100730 China

**Keywords:** Cytomegalovirus, Colitis, Intestinal obstruction

## Abstract

**Background:**

Cytomegalovirus infection manifests varying clinical characteristics and severity in diverse populations with different immune statuses. The signs and symptoms of gastrointestinal involvement are nonspecific. Here, we present a case of cytomegalovirus colitis in an immunocompetent adolescent, which manifested as intestinal pseud-obstruction.

**Case presentation:**

A 15-year-old man who had contracted novel coronavirus infection one month earlier was admitted to our hospital with fever, abdominal pain, and hematochezia. His abdomen was distended, and laboratory evaluation revealed a decrease in the blood count, an increase in inflammatory indicators and hepatic impairment. Imaging shows bowel wall thickening and dilatation of the colon. A diagnosis of intestinal infection combined with acute intestinal pseud-obstruction was made. Diarrhea persisted despite conservative treatment with empirical antibiotics. A colonoscopy was performed. Pathology confirmed cytomegalovirus infection. Ganciclovir therapy was initiated, and subsequent review showed a good recovery.

**Conclusions:**

The case was diagnosed as cytomegalovirus colitis. We reviewed the reports of 9 cases of bowel obstruction, including our own, and found that the majority of the adult patients were elderly with underlying disease. Clinical and endoscopic manifestations are typically nonspecific, and imaging shows typical signs of intestinal obstruction. The final diagnosis was confirmed by pathology. Most of them have a good prognosis. We suggest that cytomegalovirus colitis can also lead to intestinal obstruction and that viral reactivation in immunocompetent individuals may be associated with inflammatory conditions and viral coinfection, particularly with the novel coronavirus.

**Supplementary Information:**

The online version contains supplementary material available at 10.1186/s12879-024-09255-7.

Cytomegalovirus (CMV) infection is common, with approximately 83% of the global population testing seropositive [1]. People with normal immune function usually have no symptoms, or they may experience fever, mononucleosis, or hepatitis. Immunodeficient individuals are susceptible to viral reactivation, which can affect end organs, such as the lungs, central nervous system, and digestive tract. Recently, there has been a notable increase in CMV colitis among immunocompetent patients, which has garnered significant attention. In healthy individuals, the most affected part of the digestive tract is the colon, exhibiting symptoms like hemorrhage, abdominal pain, and diarrhea [2]. CMV colitis with intestinal obstruction as the primary manifestation has rarely been reported before.

## Case presentation

A 15-year-old male was admitted to the emergency room after presenting a high fever for 10 days, abdominal colic, and mucous bloody stool for 4 days. He had severe abdominal distension and a cessation of passing gas. A month ago, he was suspected of being infected with the novel coronavirus (SARS-CoV-2) due to fever and a sore throat. He took ibuprofen to defervesce. Afterward, jaundice developed. Laboratory tests in other hospitals found that transaminase levels increased, and liver injury caused by acute infection was considered, but no additional pathogenic tests were conducted. Silybin and other hepatoprotective drugs were administered. One week later, symptoms were relieved, and transaminase levels decreased. In addition, he had no other notable medical or family history.

His complete blood count showed a white blood cell count of 0.36 × 10^9^/L (Normal range(NR): 3.50–9.50 × 10^9^/L), with neutrophils accounting for 45.8% (NR: 50%-75%), lymphocytes accounting for 42.0% (NR: 20%-40%), hemoglobin 57 g/L (NR: 120-160 g/L), and platelet count of 59 × 10^9^/L (NR: 100–350 × 10^9^/L); his C-reactive protein was 267 mg/L (NR: < 8.0 mg/L). There was also hepatic impairment, with ALT was 72U/L (NR: 9-50U/L) and AST was 54U/L (NR: 15-40U/L). The stool sample showed an elevated level of white blood cells (20–30/HPF, NR: 0–1) and red blood cells (2–3/HPF, NR: 0). Abdominal computed tomography (CT) showed thickening of the rectal and sigmoid bowel wall with dilation of the proximal colon (Supplementary Fig. 1).

We diagnosed as probable intestinal infection and acute intestinal pseud-obstruction. Colonoscopy is considered risky at this time. We promptly inserted an ileus catheter and initiated empirical therapy with meropenem, vancomycin, and human immunoglobulin. His abdominal pain resolved, and his blood count and liver function gradually normalized, but diarrhea continued.

**Fig. 1:**
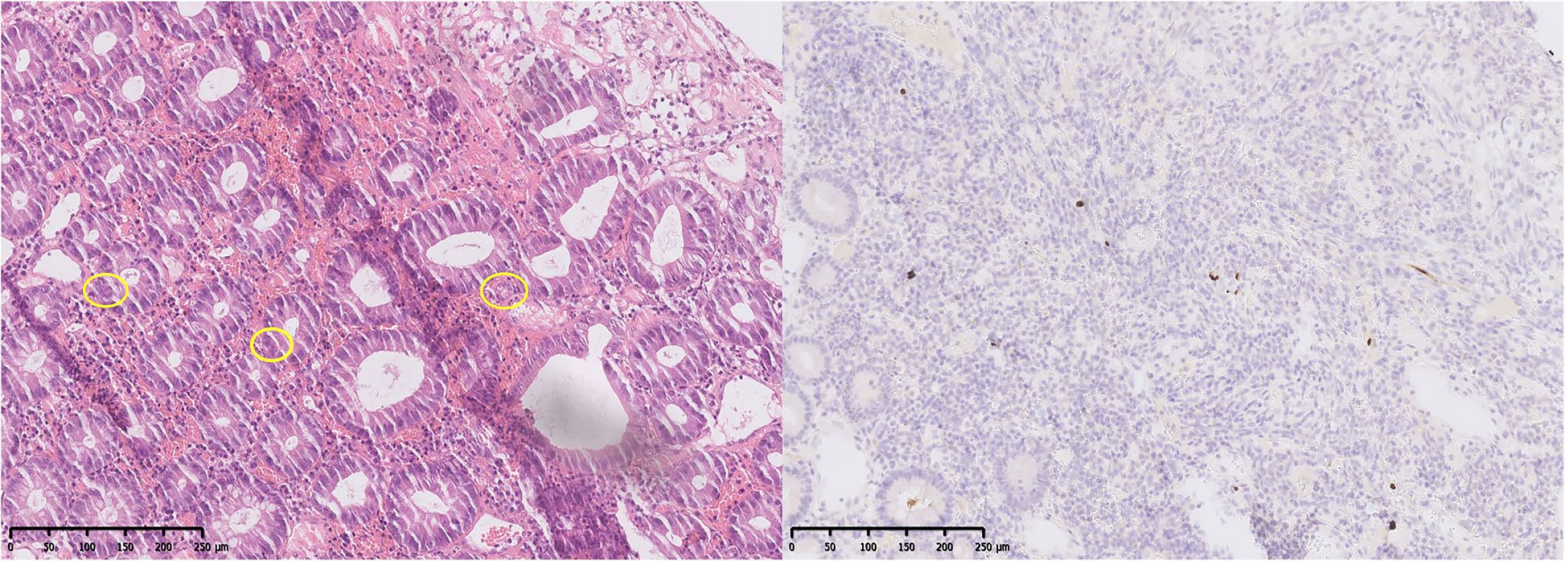
Histopathological examination of the patient. **a** HE staining, × 10 magnification. Multiple cytomegalic inclusions are present. **b** IHC staining, × 10 magnification. A positive area of CMV antigen stained brown is visible

The persistence of the symptoms compelled us to continue the search for the cause. Plasma metagenomic next-generation sequencing (mNGS) showed 50 Epstein‒Barr viruses; serum polymerase chain reaction showed that CMV copy was negative, and plasma CMV-IgG/IgM were also negative. Plasma SARS-CoV-2 IgG was positive, confirming a recent case of novel coronavirus disease (COVID-19). The test for Clostridium difficile, fungi, Mycobacterium tuberculosis, Salmonella, Shiga, rotavirus, and parasites in feces was negative. Other tests, such as antinuclear antibody, antiphospholipid antibody, and HIV tests, were negative. Seventeen days later, he was scheduled for a colonoscopy. Sigmoid colonic and rectal ulcers were observed (Supplementary Fig. 2A, B). Hematoxylin–eosin (HE) staining revealed CMV inclusion bodies, and immunohistochemical (IHC) staining demonstrated positive CMV antigen (Fig. 1), confirming colitis caused by CMV infection.

We started intravenous ganciclovir at a dosage of 500 mg/daily for 21 days. Meanwhile, due to the destruction of the superficial intestinal mucosa and persistent diarrhea, considering that inflammatory bowel disease cannot be definitely ruled out, he was also given oral mesalazine and prednisone (starting at 70 mg/daily) to repair the intestinal mucosa and reduce inflammatory reactions after consulting with the Department of Gastroenterology. During a follow-up examination three months later, his blood count and biochemical indicators were normal. Endoscopic findings revealed stenosis of colon lumen and histological signs of chronic inflammation (Supplementary Fig. 2C, D). CMV was negative for the IHC test, and his stool gradually returned to normal.

## Discussion

This is a case of CMV colitis with acute intestinal pseud-obstruction. After the catheter was inserted, the obstructive symptoms were quickly relieved. Although there was no direct serological evidence, CMV infection was subsequently confirmed through pathological examination. However, this case occurred in an immunocompetent adolescent who was infected with the novel coronavirus one month ago. Liver injury before onset is considered most probably as one of the manifestations of CMV reactivation. After extensive evaluation, no evidence of existing immunodeficiency leading to CMV disease was found.

A total of 9 cases [3–10], including our case, were identified by searching PubMed using the keywords "cytomegalovirus" and "colitis" and reviewing relevant literature citations to retrieve previously reported cases of CMV colitis with intestinal obstruction. Patients with inflammatory bowel disease, AIDS, organ transplantation, and treatment with steroids or immunosuppressive agents were excluded (Supplementary Table 1). Of these, 6 (66.7%) were male. The average age was 61 years (range: 15–91), and 66.7% of the participants were over 60 years old. 6 (66.7%) of them had multiple co-morbidities, including chronic constipation, diabetes, chronic obstructive pulmonary disease, and other complications. Most of the cases were diagnosed through pathology. 7 of them were treated with ganciclovir, and a total of 2 (22.2%) people died.

The retrospective analysis also revealed that the most common symptoms were fever, abdominal pain, and diarrhea. The endoscopic examination revealed mostly ulceration, while imaging findings showed dilated intestines. M. Paparoupa et al. [10] and Dinesh et al. [5] reported cases of mechanical obstruction caused by fibrotic stenosis, thickening and edema of the bowel wall. In the present case and in the remaining cases, the location of the obstructive lesion was not identified, and paralytic ileus caused by toxic megacolon or Ogilvie syndrome was considered. The most common sites involved are the sigmoid colon and rectum. Based on the results of our study, we believe that CMV colitis should increase the rare clinical presentation of intestinal obstruction, even in a population with normal immunity. For patients diagnosed as CMV colitis with normal immunity, clinicians should observe abdominal symptoms and signs in clinical practice, and be alert for acute intestinal obstruction. Similarly, the presence of CMV should be reasonably suspected in patients with intestinal obstruction. Analysis showed that conservative treatments, such as ganciclovir and placement of obstruction catheters, were usually effective for CMV colitis presenting with intestinal obstruction, without the need for surgery.

It is believed that CMV latent infection exists in early myeloid cells, particularly CD34^+^ hematopoietic stem cells, which can be reactivated to infect parenchymal cells of various organs [11]. Viremia is crucial for pathogenesis, and there is a threshold relationship between the viral load controlled by the body's immunity and the occurrence of end-organ invasion [12]. Therefore, virus reactivation and corresponding diseases can easily occur when immunity is inhibited or damaged. However, immunocompetent people are not always unaffected. A retrospective analysis conducted by Yoon et al. [2] revealed that the majority of immunocompetent patients diagnosed with CMV gastroenteritis were elderly (74.4%) and had underlying chronic conditions (79.1%), which is consistent with our study. Immunity is affected by both aging and the basal state, so it is reasonable to assume that CMV disease may be rare in truly immunocompetent young patients. The patient's infection occurred after COVID-19, prompting us to further explore the interaction between these two viruses. An increased incidence of herpes virus reactivation has been observed in patients with COVID-19 [13]. In addition to being associated with inflammatory responses and steroid use, SARS-CoV-2 infection results in a reduction in lymphocytes and upregulation of T-cell apoptotic processes [14, 15]. The ACE-2 receptor of SARS-CoV-2 entering cells is highly expressed in gastrointestinal epithelial cells. The immune regulation of the lung-gut axis indirectly affects gastrointestinal endothelial cells through pulmonary infection and is susceptible to CMV [16]. These are all proposed as possible mechanisms of CMV reactivation after SARS-CoV-2 infection. The specific mechanisms of virus-immune system interactions require further investigation and validation in large cohorts.

## Conclusions

Cytomegalovirus disease involving the digestive tract can occur in immunocompetent hosts. Aside from abdominal pain, it can also manifest as intestinal obstruction, and generally, conservative treatment is effective. Early recognition of CMV colitis presenting with intestinal obstruction helps to avoid unnecessary disease prolongation and improves prognosis. The reactivation of cytomegalovirus is associated with inflammation in healthy individuals, and novel coronavirus coinfection is considered to be one of the potential contributing factors.

### Supplementary Information

**Additional file 1: Supplementary Figure 1.** (A, B): Abdominal computed tomography. (A) The distended transverse colon. (B) Thickening of the rectal and sigmoid colon wall with peripheral inflammatory exudation.

**Additional file 2: Supplementary Figure 2.** (A, B, C, D): The first colonoscopy and three months later, sigmoid colon and rectum. (A, B) 17 days after being hospitalized. Thickening and edema of the intestinal wall, a smaller intestinal cavity, and deep ulcers were observed. (C, D) 3 months after discharge. A narrow intestinal cavity and inflammatory polyps are visible, but the ulcer has improved.

**Additional file 3: Supplementary Table 1. **Clinical characteristics of 9 cases of CMV enteritis with intestinal obstruction.

## Data Availability

All data generated or analyzed during this study are included in this published article.

## Abbreviations

CMV: Cytomegalovirus
CT: Computed tomography
mNGS: Metagenomic next-generation sequencing
HE: Hematoxylin–eosin
IHC: Immunohistochemical
NR: Normal range
