# Supplementary figures and images for: Cytomegalovirus colitis as intestinal obstruction in an immunocompetent adolescent: a case report and literature review
Source: BMC Infect Dis. 2024 Apr 1;24:365. doi: 10.1186/s12879-024-09255-7 (PMC10983691; doi:10.1186/s12879-024-09255-7)

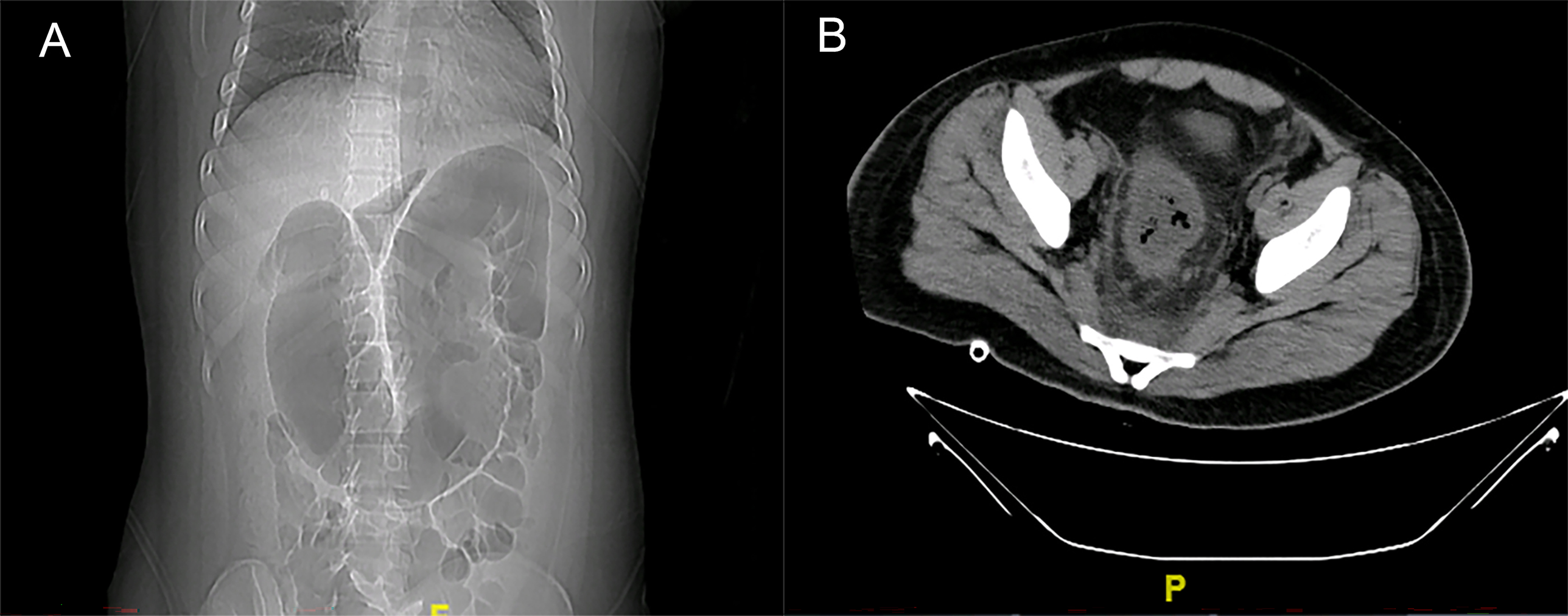

Supplement: Supplementary file 1 — Additional file 1: Supplementary Figure 1. (A, B): Abdominal computed tomography. (A) The distended transverse colon. (B) Thickening of the rectal and sigmoid colon wall with peripheral inflammatory exudation. [file 12879_2024_9255_MOESM1_ESM.jpg]
