# Supplementary material for: Cytomegalovirus colitis as intestinal obstruction in an immunocompetent adolescent: a case report and literature review
Source: BMC Infect Dis. 2024 Apr 1;24:365. doi: 10.1186/s12879-024-09255-7 (PMC10983691; doi:10.1186/s12879-024-09255-7)
Supplement: Supplementary file 3 — Additional file 3: Supplementary Table 1. Clinical characteristics of 9 cases of CMV enteritis with intestinal obstruction. [file 12879_2024_9255_MOESM3_ESM.docx]

**SUPPLEMENTARY TABLE 1.**

Clinical characteristics of 9 cases of CMV enteritis with intestinal obstruction

|  | | | | | | | | | |
| --- | --- | --- | --- | --- | --- | --- | --- | --- | --- |
| Ref | Gender | Age(yrs.) | Comorbidities | Clinical manifestations | Image features | Endoscopic finding/ Operative finding | Pathological diagnosis | Treatment | Outcome |
| Machens et al. | M | 75 | Multiple injuries caused by traffic accident | Fever, bloody diarrhea | Not reported | Ulcer | Yes | Colectomy | Died(Due to multiple organ failure) |
| Lin et al. | M | 70 | Parkinson’s disease, benign prostatic hypertrophy, chronic constipation | Abdominal distention, diarrhea | Dilated bowel | Ulcer, polyp | Yes | Ganciclovir | Improved |
| Dinesh et al. | M | 65 | None | Colicky pain, emesis, constipation | Dilated bowel, enhancing short segment stricture at the descending colon-sigmoid colon junction | Ulcer | Yes | Colectomy | Improved |
| Cho et al. | M | 70 | None | Abdominal pain, constipation, hematochezia | Dilated bowel | Ulcer, mucosal edema | Yes | Ganciclovir | Improved |
| Charatcharoenwitthaya et al. | F | 38 | Uterine curettage | Fever, diarrhea | Dilated bowel | Ulcer | Yes | Ganciclovir | Improved |
| Kim et al. | F | 48 | Chronic constipation | Abdominal pain, constipation | Dilated bowel; edematous colonic wall thickening in the sigmoid colon(8 months later) | Ulcer | Yes | Ganciclovir, oral laxatives | Improved |
| Lagarto et al. | F | 91 | Depression, hypertension, type2 diabetes mellitus | Abdominal pain and bloating, diarrhea | Dilated bowel | Ulcer | Yes | Ganciclovir, oral valganciclovir | Died |
| M. Paparoupa et al. | M | 77 | COPD, laparoscopic cholecystectomy | Ileus symptoms, hematochezia | Not reported | Diverticula, persisting stenosis | No | Ganciclovir | Improved |
| Present Case | M | 15 | None | Abdominal pain, mucopurulent bloody diarrhea | Dilated bowel, thickening of colon walls | Ulcer | Yes | Ganciclovir | Improved |
